# Correction

**DOI:** 10.1080/13814788.2025.2610158

**Published:** 2026-01-29

**Article title:** Towards consensus: The need for standardised definitions in Long (post) COVID care in 34 European countries

**Authors:** Raquel Gomez-Bravo, Sandra León-Herrera, Marina Guisado-Clavero, Ileana Gefaell, Xenia Wostmann, Nathalie Wössner, Shlomo Vinker, Francesca Vassallo La Ferla, Erva Kırkoç Üçüncü, Georgi Tsigarovski, Péter Torzsa, Kadri Suija, Aleksander Stepanović, Theresa Sentker, Anna Segernäs, Bohumil Seifert, Marta Sánchez-Castro, Jochen G. Schneider, Anna Repovská, Ferdinando Petrazzuoli, Davorina Petek, Abel Perjes, Naldy Parodi López, Ana Luisa Neves, Katarzyna Nessler, Jean Muris, Achim Mortsiefer, Sarah Moreels, Tatjana Meister, Pekka Mäntyselkä, Liubovė Murauskienė, Heidrun Lingner, Anna Krztoń-Królewieckaee, Milena Kosticff, Büsra Çimen Korkmaz, Snezana Knezevic, Stylianos Kazakos, Vasilis Karathanos, Ivanna Shushman, Oksana Ilkov, Kathryn Hoffmann, Bruno Heleno, Miroslav Hanževački, Dragan Gjorgjievski, Thomas Frese, Marta Fournier, Louise Fitzgerald, Sabīne Feldmane, Marina Dotsenko, Philip-Richard Domeyer, Daniel Croucher, Vojtech Cerny, Jako S. Burgers, Elena Brutskaya-Stempkovskaya, Carmen Iliana Busneag, Nicola Buono, Sherihane Bensemmane, Sabine Bayen, Maria Bakola, Radost Assenova, Limor Adler, Sara Ares-Blanco and María Pilar Astier-Peña

**Journal:** *European Journal of General Practice*

**Bibliometrics:** Volume 31, Number 1, 2535618

**DOI:** https://doi.org/10.1080/13814788.2025.2535618

The following references were incorrectly submitted into production:

[31] Luntamo T, Aromaa M, Kallioinen M, et  al. Functional disorders and post-viral syndromes in primary care: lessons for long COVID. Lancet Reg Health Eur. 2024;38:101140. doi: 10.1016/j.lanepe.2024.101140.

[32] Rando HM, Bennett TD, Byrd JB, et  al. Challenges in defining long COVID: striking differences across literature, electronic health records, and patient-reported information. Lancet. 2024;403(10427):1234–1239. doi: 10.1016/S0140-6736(24)00623-8.

[33] Theoharides TC. Long COVID: a neuroimmune disorder with potential mast cell involvement. Neuroimmunomodulation. 2024;31(1):1–9. doi: 10.1159/000541741.

[34] Roveta F, Tzovaras D, Barbone F. Managing post-viral symptoms in primary care: the case for integrated, symptom-based approaches. Lancet Respir Med. 2022;10(11):1045–1053. doi: 10.1016/S2213-2600(22)00501-X

The correct references are as follows:

[31] Toussaint A, Weigel A, Löwe B, EURONET-SOMA group. The overlooked burden of persistent physical symptoms: a call for action in European healthcare. Lancet Reg Health Eur. 2025;48:101140. doi: 10.1016/j.lanepe.2024.101140.

[32] Löwe B, Toussaint A, Rosmalen JGM, et al. Persistent physical symptoms: definition, genesis, and management. Lancet. 2024;403(10444):2649–2662. doi: 10.1016/S0140-6736(24)00623-8.

[33] Chou R, Herman E, Ahmed A, et al. Long COVID definitions and models of care: a scoping review. Ann Intern Med. 2024;177(7):929–940. doi: 10.7326/M24-0677.

[34] Engelmann P, Reinke M, Stein C, et al. Psychological factors associated with Long COVID: a systematic review and meta-analysis. EClinicalMedicine. 2024;74:102756. doi: 10.1016/j.eclinm.2024.102756.

